# *Trypanosoma* Infection Rates in *Glossina* Species in Mtito Andei Division, Makueni County, Kenya

**DOI:** 10.1155/2015/607432

**Published:** 2015-11-04

**Authors:** Daniel Mutiso Nthiwa, David O. Odongo, Horace Ochanda, Samoel Khamadi, Bernard M. Gichimu

**Affiliations:** ^1^Department of Biological Sciences, Embu University College, P.O. Box 6, Embu 60100, Kenya; ^2^School of Biological Sciences, University of Nairobi, P.O. Box 30197, Nairobi 00100, Kenya; ^3^Kenya Medical Research Institute, Center for Virus Research, P.O. Box 54840, Nairobi 00200, Kenya; ^4^Department of Agricultural Resource Management, Embu University College, P.O. Box 6, Embu 60100, Kenya

## Abstract

African Animal Trypanosomiasis (AAT) transmitted cyclically by tsetse fly (*Glossina *spp.) is a major obstacle to livestock production in the tropical parts of Africa. The objective of this study was to determine the infection rates of trypanosomes in *Glossina *species in Mtito Andei Division, Makueni County, Kenya. Tsetse fly species, *G. longipennis* and *G. pallidipes*, were trapped and DNA was isolated from their dissected internal organs (proboscis, salivary glands, and midguts). The DNA was then subjected to a nested PCR assay using internal transcribed spacer primers and individual trypanosome species were identified following agarose gel electrophoresis. Out of the 117 flies trapped in the area 39 (33.3%) were teneral while 78 (67%) were nonteneral. *G. pallidipes *constituted the largest percentage of 58% while *G. longipennis *were 42%. The overall trypanosomes infection rate in all nonteneral *Glossina *spp. was 11.53% with *G. longipennis* recording the highest infection rate of 23.08% while *G. pallidipes *had an infection rate of 5.77%. *T. vivax *was the most infectious (10.26%) compared to *T. congolense *(1.28%). Mean apparent densities were strongly positively correlated with infection rates (*r* = 0.95) confirming the importance of this parameter as an indicator of AAT transmission risk.

## 1. Introduction

African Animal Trypanosomiasis (AAT) is a serious livestock disease caused by a Kinetoplastida protozoan parasite of the genus* Trypanosoma* and transmitted cyclically by tsetse (*Glossina*) and mechanically by other hematophagous flies such as* Tabanus*,* Haematopota*,* Stomoxys*, and* Chrysops* [1, 2]. It is a major constraint of livestock production in sub-Saharan Africa as it prevents full use of the land to feed the rapidly increasing human population [3]. Subsequently, this contributes to poverty, hunger, and underdevelopment in the affected areas [4]. This disease is therefore a major impediment to agricultural and economic advancement in the endemic areas [5, 6]. AAT and its vectors occur in large areas of sub-Saharan Africa and its occurrence parallels that of the biological vector [7, 8]. The epidemiology and effects of this disease on livestock especially cattle production are determined largely by the prevalence and distribution of the disease and its vectors in the affected areas and also the proportion of infected flies in the tsetse population [9]. The ecodistribution of tsetse is determined by climate, presence of vegetation, water, and presence of blood meals (humans and animals) but tsetse are mostly found in rural areas [10].

The economic losses due to AAT are approximated to be over 1.5 billion dollars annually [11]. Tsetse infest about 10 million km^2^ of fertile land in Africa spread across 36 countries with approximately 7 million km^2^ of the infested land being suitable for mixed agriculture if the disease was controlled [11]. Out of 165 million cattle found in Africa only 10 million are found within the tsetse belt due to the disease constraint and these are the lowest producing breeds [12]. The Kenyan economy is largely agricultural-based, with the sector accounting for about 24% of the Gross Domestic Product (GDP) and 70% of the employment [13]. Only 7% of Kenyan land is suitable for crop production and a further 5% can sustain crops in years of adequate rains [13]. The rest of land is arid and semiarid and constitutes the range lands which are not suitable for crop production but rather good for livestock production [13, 14].

Mtito Andei is located in Makueni County about 290 km South East of Nairobi. The area is comprised mainly of arid and semiarid lands and livestock rearing is the mainstay of this region [14]. This area has a history of animal trypanosomiasis as it is infested with* G. pallidipes*,* G. longipennis*, and* G. brevipalpis* [14, 15]. The area was not inhabited until the 1930s due to its low agricultural potential and heavy tsetse infestation. Assessment of prevalence vector hotspots and infection status can be instrumental in making decisions with regard to the formulations of suitable tsetse and trypanosomiasis control measures. However, there is no accurate information that exists in regard to the current prevalence of trypanosomiasis in Mtito Andei. This is because previous data has been collected through microscopy on blood smears, dissection, and microhematocrit techniques which are less sensitive [16, 17]. Further, these methods cannot identify immature and mixed infections, subsequently resulting to low detection levels [18]. This study was aimed at providing additional information on the prevalence of trypanosomes in* Glossina* species in Mtito Andei.

## 2. Materials and Method

### 2.1. Description of the Study Site

**Figure 1:**
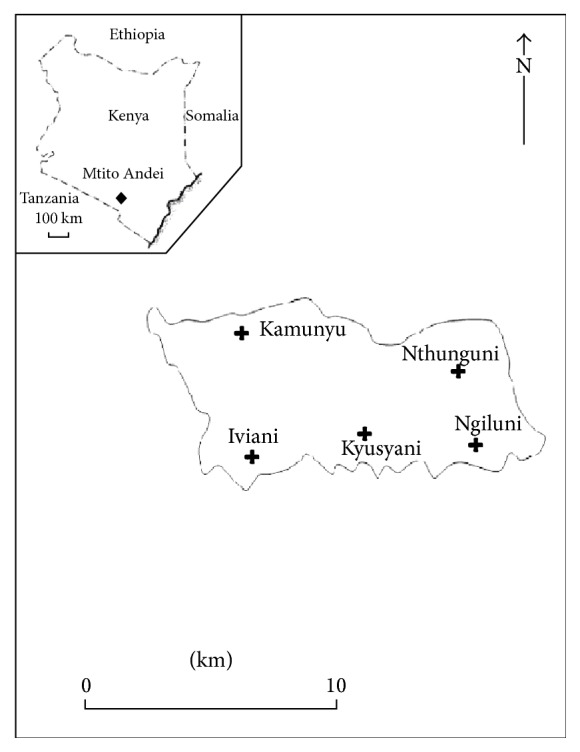
Map of Mtito Andei showing the georeferenced trapping sites.

This study was carried out in Mtito Andei Division situated in Makueni County about 290 km South East of Nairobi. The study area was divided into five villages, namely, Nthunguni, Iviani, Kyusyani, Ngiluni, and Kamunyu (Figure 1). The altitude of the area varies from 600 m to 1100 m above sea level. The area is characterized by marginal agricultural lands of Savannah grassland with mostly low lying, gently eastward sloping plains occasionally broken by seasonal and perennial rivers [20]. The climate of this area is typically semiarid with low and unreliable rainfall averaging 600 mm annually and annual average temperature of 23°C [20]. Rainfall is bimodal with long rains occurring from March to May and short rains from November or December to early January. The highest mean temperatures between 32°C and 33°C prevail in February to March [20].

### 2.2. Study Design

A cross-sectional study using simple random sampling technique was conducted to determine the infection rates of trypanosomes in* Glossina* species in Mtito Andei Division. Sample size was determined using the formula (*n* = *Z* ^2^ *PQ* ^2^/*d* ^2^), described in [21], where *n* is the minimum sample size required, *Z* is the confidence interval, *P* is the expected prevalence in proportion from the study, *Q* is 100% minus *P*, and *d* is the desired precision taken at 0.05.

### 2.3. Collection and Identification of Tsetse

**Figure 2:**
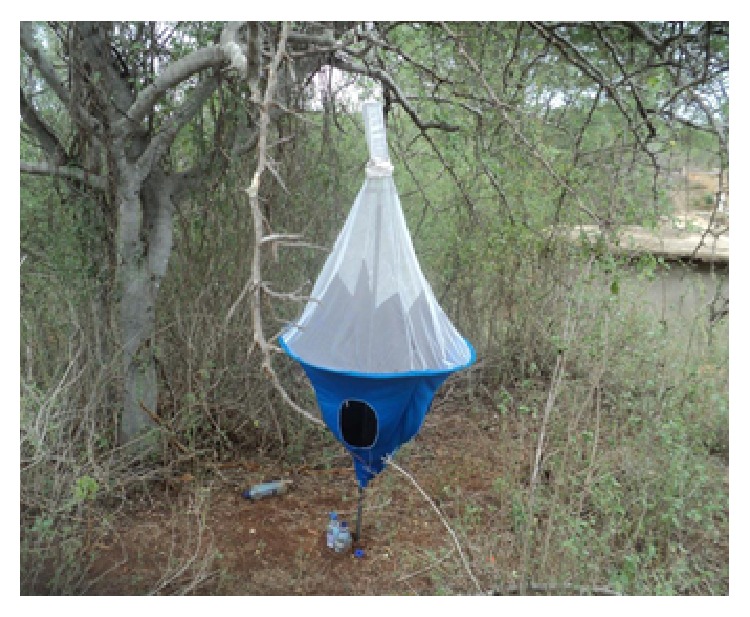
A biconical trap baited with cow urine and acetone to enhance trapping efficiency.

Tsetse trapping was done between April and May 2012. This period was chosen because it is a rainy season and therefore more abundant flies were expected [1]. Trapping was done using 5 biconical traps in each village which were deployed near Mtito Andei River and Athi River and in the nearby vegetation. The traps were set at georeferenced sites and baited with acetone and cow urine to increase trapping efficiency [22]. The traps were set at regular intervals of 100 meters along various vegetation types and under shade to avoid undue fly mortality due to heat (Figure 2). To prevent the ascent of ants on the poles towards the collecting cages, each pole was smeared with grease. All trapped flies were counted; their sex was determined and identified to species level using conventional identification keys before dissection [23].

### 2.4. Determination of Apparent Density (AD)

Apparent density (AD) is an estimation of flies' density and is given in terms of the number of flies caught per trap per day (*FTD*). It was calculated using the following formula:

(1) $$\begin{array}{c} FTD=\frac{\sum F}{T\times D}, \end{array}$$

where ∑⁡*F* is the number of total flies, *T* is the number of functioning traps, and *D* is the number of days for which traps were operational [1].

The average AD for each trapping site was then calculated to obtain the data on tsetse distribution in the area.

### 2.5. Identification of Trypanosomes in* Glossina* spp. 

#### 2.5.1. Tsetse Dissection

The flies were immobilized using ethyl acetate placed in a cotton wool at the bottom of a specimen tube. Dissection was carried out on freshly immobilized flies as trypanosomes are less likely to be found in dead dried flies [23]. A total of 78 nonteneral flies were dissected from which midgut, salivary glands, and proboscis were removed and analyzed under a microscope for the presence of trypanosomes. Nonteneral flies were identified by the presence of dark or brown colour on their abdomen which indicated the last blood meal [23]. Freshly immobilized flies were surface sterilized by brief immersion in 70% ethanol, then dry blotted on clean tissue paper, and then dissected in normal saline [22]. To avoid cross contamination, the dissecting instruments were sterilized by immersion in 3–5% (w/v) sodium hypochlorite for approximately 2 minutes, followed by extensive rinsing in distilled water and later final immersion in normal saline [22]. Dissected organs (midgut, proboscis, and salivary glands) from each fly were transferred into 1.5 mL microcentrifuge tube containing 120 *μ*L of tissue lysis buffer (from Qiagen DNeasy Blood and Tissue Kit). The tissues were then stored at −20°C until further processing.

#### 2.5.2. Trypanosomes' DNA Extraction

DNA was extracted using the Qiagen DNeasy blood and tissue kit and according to the manufacturer's instructions. Twenty (20) microliters (*μ*L) of proteinase K was added to the dissected tissues and incubated at 56°C overnight in a thermocycler until the tissue was completely digested. The lysate was vortexed for 15 sec and 200 *μ*L of buffer AL was added to the sample followed by vortexing to mix the contents. Two hundred (200) *μ*L of absolute ethanol was added and mixed thoroughly by further vortexing before pipetting the mixture into DNeasy mini spin columns placed in a 2 mL collection tube. The columns were centrifuged at 8000 rpm for 1 minute and the flow through discarded together with the collection tube. The DNeasy mini spin columns were placed in a new 2 mL collection tube and 500 *μ*L of wash buffer AW1 was added. It was then centrifuged at 8000 rpm for 1 minute and the flow through together with the collection tube discarded. The spin column was again placed in a new collection tube and 500 *μ*L of wash buffer AW2 was added. The column was centrifuged at 14,000 rpm for 3 minutes. The flow through and the collection tubes were discarded and the spin columns placed in a clean 1.5 mL microcentrifuge tube followed by addition of 30 *μ*L of elution buffer (AE buffer). The column was incubated at room temperature for 1 minute and centrifuged for 1 minute at 8000 rpm to elute the bound DNA. DNA extracted from samples was quantified using a nanodrop spectrophotometer (Thermo Fisher Scientific Inc.) and its purity was determined by measuring the intensity of absorbance at wavelengths 260 and 280 nanometers.

#### 2.5.3. PCR Amplification and DNA Analysis

The PCR oligonucleotide primers used are shown in Table 1. The primers were synthesized by Bioneer Company and were provided by International Livestock Research Institute (ILRI), Kenya.

**Figure 3:**
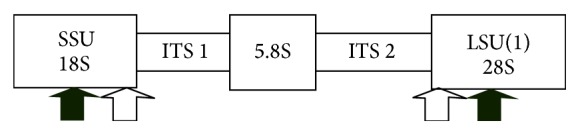
Schematic diagram of rDNA showing ITS 1 and ITS 2 annealing positions [19].

These primers were designed to amplify the internal transcribed (ITS) region of ribosomal DNA (rDNA), a region which varies in size within trypanosome species, except for members of Trypanozoon, and are capable of differentiating trypanosomes based on their amplified fragments. ITS 1 and ITS 2 (outer primers) amplify the conserved regions of rDNA, small subunit (SSU), and large subunit (LSU), represented by black arrows in Figure 3 while ITS 3 and ITS 4 (inner primers) amplify the noncoding spacer regions of rDNA and are represented by white arrows.

All PCR amplifications were performed in 30 *μ*L reaction volumes containing final concentrations of 1x Invitrogen PCR buffer, 3.5 mM MgCl_2_ (Promega), 0.2 mM dNTPs mix, 1.25U/*μ*L Invitrogen Taq, 100 ng/*μ*L of each forward and reverse primer, and 5 *μ*L of trypanosome DNA as template. A set of purified genomic DNA of* T. brucei brucei* (T.b.b.),* T. congolense* Kilifi (T.c.k.), and* T. congolense* Savannah (T.c.s.) (strain IL1180) were included as positive controls during all PCR assays and nuclease free water as negative control. The outer primers (ITS 1 and 2) were used in the first round of reaction. The cycling conditions were as follows: 1 cycle of 95°C for 7 minutes as initial denaturation followed by 35 cycles of 94°C for 1 minute, 55°C for 1 minute, and 72°C for 2 minutes. The final extension was done at 72°C for 5 minutes. The PCR was carried out on a Bio-Rad thermocycler. In the second round of reaction, the outer primers were substituted with inner primers (ITS 3 and 4) and 5 *μ*L of round one PCR product was used as DNA template. The cycling conditions for the second round of reaction were as described for the primary amplification. To analyze the amplicons, 10 *μ*L of the PCR product was resolved in a 2% agarose gel at 80 volts for 45 minutes and the gel was visualised under UV transilluminator following ethidium bromide staining.

### 2.6. Data Analysis

The infectious trypanosomes were identified by comparing the molecular sizes of their DNA fragments with the documented band sizes of trypanosome species [19]. The data was then used to calculate the infection rates and prevalence of the trypanosomes on* Glossina* spp. Conventional data on vector distribution, apparent densities, and infection rates from the five sites was subjected to one-way analysis of variance (ANOVA) using SPSS version 16.0 software at 95% confidence interval. Chi-square test was done to determine the association of infection rates with fly's sex and vector species. A correlation analysis was also done to establish whether there was a significant relationship between infection rates and apparent densities.

## 3. Results

### 3.1. Vector Distribution

**Figure 4:**
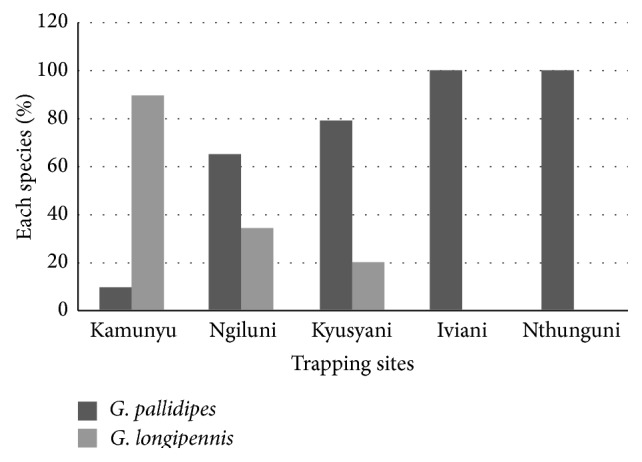
Distribution of* Glossina* species captured from study sites in Mtito Andei Division.

Out of the 117 flies trapped in the area,* G. pallidipes* constituted the largest percentage of 58% while* G. longipennis* had 42%. Among the trapped tsetse flies, 39 (33.3%) were teneral while 78 (67%) were nonteneral. In Kamunyu area,* G. longipennis* was the most prevalent species while* G. pallidipes* dominated Ngiluni, Kyusyani, Iviani, and Nthunguni (Figure 4).

**Figure 5:**
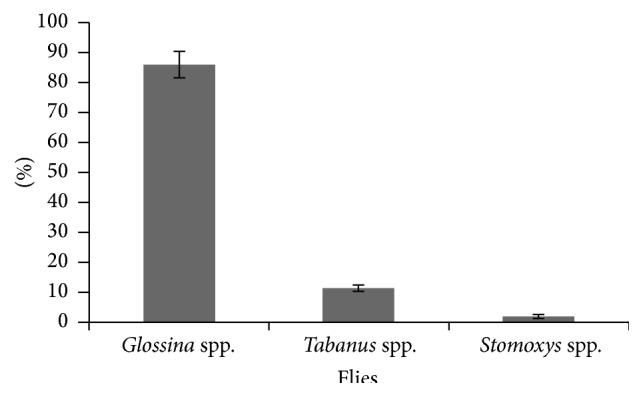
Relative distribution of total fly catch.

Apart from* Glossina* spp., the area was also inhabited with mechanical flies such as* Tabanus* spp. and* Stomoxys* spp.* Tabanids* were highly predominant over* Stomoxines* in all the trapping sites with an overall fly trap density (FTD) of 0.11 while* Stomoxys* spp. had FTD of 0.02. Of the total fly catch,* Glossina* spp. were the most prevalent in all the tapping sites while* Stomoxys* spp. constituted the lowest percentage and were recorded only in Kyusyani area (Figure 5).

### 3.2. Apparent Densities (AD)

**Figure 6:**
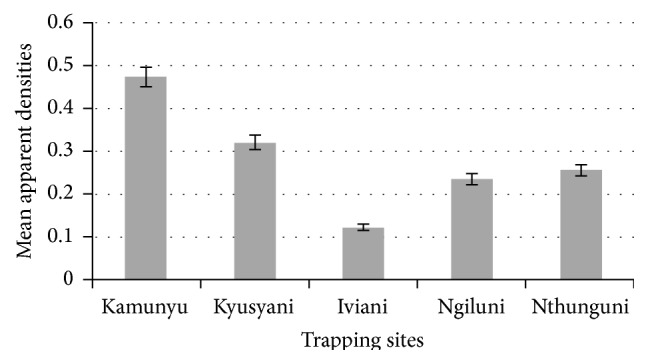
Comparison of mean apparent densities within the five trapping sites.

There was no significant difference in the apparent densities (AD) of* Glossina* species captured across the five trapping sites which ranged from 0.1 to 0.5 depending on the locality and averaged 0.3 flies per trap per day (Figure 6).

### 3.3. Identification of Trypanosomes in* Glossina* spp.  

The expected band sizes of trypanosomes on amplification using ITS primers are shown in Table 2. Molecular identification of trypanosomes* T. vivax* and* T. congolense* Forest in* Glossina* spp. was successful as their DNA amplicons matched the expected sizes of 611 bp and 1513 bp, respectively (Table 2). The amplified fragments for positive controls of* T. brucei brucei*,* T. congolense* Kilifi, and* T. congolense* Savannah were also within the expected sizes (Table 2).

**Figure 7:**
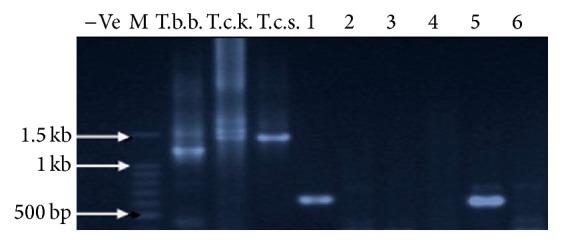
An image of an agarose gel showing PCR amplified DNA fragments using ITS nested PCR primers.

Figure 7 shows a sample of electrophoresis product of successfully amplified DNA fragments of trypanosome species* T. vivax* (samples 1 and 5) and the positive controls* T. brucei brucei* (T.b.b.),* T. congolense* Kilifi (T.c.k.), and* T. congolense* Savannah (T.c.s.). “M” represents 100 base pair molecular marker (Promega) while 6 is the negative control. Samples 2, 3, and 4 were negative for trypanosomes.

### 3.4. Infection Rates

Out of 78 flies analyzed using nested PCR and gel electrophoresis, the prevalence of trypanosomes infection in* G. pallidipes* and* G. longipennis* was 5.77% and 23.08%, respectively (Table 3). The overall infection rate in all* Glossina* spp. was recorded as 11.53%. There was a significant (*p* < 0.05) difference in the trypanosomes infection rate among* Glossina* species. Infections with* T. vivax* were the most prevalent (10.26%) in both species of tsetse compared to* T. congolense* with a prevalence of 1.28% (Table 3).

There was significant (*p* < 0.05) difference in infection rates among the trapping sites with Kamunyu recording the highest rate of 6.41% followed by Kyusyani at 2.56%. Ngiluni and Iviani recorded similar infection rates of 1.28% while Nthunguni had an infection rate of 0%.

A correlation analysis between apparent densities and infection rates showed a strong positive relationship with a correlation coefficient of 0.95. For instance, Kamunyu area recorded the highest apparent density as well as the highest infection rates.

There was a significant difference (*p* < 0.05) in* T. vivax* and* T. congolense* infections among male and female flies. For both* T. vivax* and* T. congolense*, the infections were higher in males than in females with an overall prevalence of 13.95% and 8.57%, respectively (Table 4).

## 4. Discussion

### 4.1. Vector Distribution in Mtito Andei

This study found that there was variation in the distribution of* G. pallidipes* and* G. longipennis* in the area.* G. longipennis* was the only fusca group of flies trapped in Mtito Andei and is known to occur in other Eastern Africa countries such as Uganda, Tanzania, and Ethiopia [2, 24]. This species is found in drier areas more than any other tsetse of fusca group because the puparium contains large water reserves and a low permeability of its pupal membranes which helps it to survive harsh environmental conditions [25, 26]. The typical habitats for this fly are dry thorn bush riverline thickets near acacia woodlands and the resting sites are preferably shady sites of tree trunks, logs, and underside of branches of multistemmed trees [24]. Mtito Andei provided all these ecological conditions hence suitable for this fly.

*G. pallidipes* is a morsitans group of flies which is widely distributed in East Afica and is a major vector of animal trypanosomiasis [27]. It was mostly found in areas close to Mtito Andei River and Athi River as it prefers dense evergreen vegetation, heavier shade, and humid habitats which are close to riverline thickets [22]. It is also present in other Eastern African countries such as Uganda, Ethiopia, and Somalia [2, 28]. Previous studies have shown that* G. pallidipes* might be a vector of* Trypanosoma b. rhodesiense* causing Human African Trypanosomiasis (HAT) in Western Kenya and Busoga District of Uganda, as this parasite has been isolated from the vector [24, 27].

Among the mechanical vectors found in Mtito Andei,* Tabanids* were highly predominant over* Stomoxines* in all the trapping sites with the latter being recorded only in Kyusyani area. This finding corroborates the report detailed in [1] that the species composition of mechanical flies is strongly dependent on site and substantial differences in environmental and climatic conditions of the sampled area. Similar observations were also made in other areas such as Brazil [29]. The presence of mechanical flies in the area cannot be underestimated as they have been shown to mechanically transmit* T. vivax* and* T. evansi* [30] and can be responsible for seasonal epidemic patterns in low tsetse density areas [1, 31].

### 4.2. Apparent Densities of* Glossina* spp. 

This study did not show significant difference in the apparent densities (AD) of* Glossina* species captured across the five trapping sites. Despite the low mean AD recorded for tsetse in this area (0.3 flies per trap per day), it is possible that tsetse serve both as biological transmitters and reservoirs of the parasite while the mechanical vectors amplify it during high density periods. Other than amplifying the parasite, the mechanical flies can introduce it to nonendemic areas through immigration.

### 4.3. Trypanosome Infection Rates

This study recorded low overall trypanosome infection rates of 11.53%. Similar results were obtained in the Kenyan Coast in a study that reported trypanosome infection rates of 12.62%, 7.24%, 9.50%, 7.86%, and 7.97% in Ukunda, Diani, Muhaka, Shimba hills, and Mwalewa, respectively [32]. A comparable study in Mouhoun River, Burkina Faso, reported overall infection rates of 10% in 1423 dissected flies screened for trypanosome infections [33]. Further, infection rates for* T. congolense* and* T. vivax* were recorded at 7.6% and 2.25%, respectively. Previous studies have also showed low prevalence of trypanosome infections (about 10%) in the field caught flies and associated this with interactions of various suppressive factors in the generally susceptible fly population [34]. In addition, there are a number of barriers to both establishment and maturation process of trypanosomes and thus only a small proportion of these infections reach maturity [24]. Further, these trypanosomes may lack differentiation trigger or may be inhibited by the vector's immune response [35, 36].

The overall trypanosomes infection rates in* G. longipennis* were significantly higher than in* G. pallidipes.* However, in Coastal Kenya, higher infection rates were reported in* G. pallidipes* (5.7%) than in* G. longipennis* (0.2%) [37]. The difference in infection rates in the two tsetse species in Mtito Andei could be due to variation in feeding preferences, environmental factors, and host range differences [11, 33]. The number of parasites available to infect tsetse, parasite infectivity to tsetse, and the strain or subspecies have also been found to affect infection rates in tsetse [38]. Moreover, the nutritional status of the tsetse at the time of infective blood meal can also affect their ability to acquire trypanosome infections [9]. In addition, low vectorial capacity has also been reported and is attributed to higher levels of attacin expression in the proventriculus and midgut [39, 40]. This kind of trait has been reported in* G. pallidipes* and it could explain disparity of infection rates between* G. pallidipes* and* G. longipennis* [34]. Within species, infection rates were also variable and this was attributed to individual host factors. For instance, the vulnerability of flies to* T. brucei* infections was shown to be due to maternally inherited features which are associated with the presence of intracellular rickettsia-like organisms (RLOs) [41]. Tsetse carrying these RLOs and other simultaneous infections such as bacteria, fungi, and virus in the midgut were more likely to be infected with trypanosomes than those without [41–43].

*T. vivax* infection was more prevalent in all trapping sites than* T. congolense*. Similar results were reported in the Luangwa Valley, Zambia [44]. However, in Côte d'Ivoire,* T. congolense* was found to be more infectious (90%) compared to* T. simiae* and* T. vivax* in 50 of 139 microscopically positive flies that were further analyzed using species specific primers for* T. congolense* subtypes,* T. simiae*, and* T. vivax* [45]. The higher* T. vivax* infections can be ascribed to short developmental lifecycle of about 10 days which is entirely completed within the proboscis. For* T. congolense* and* T. brucei* development period has been estimated to be 14 days and 30 days, respectively [46]. Further, compared to proboscis, tsetse midgut is a hostile environment which contains midgut lectins which are able to kill trypanosomes* in vivo* [47]. Midgut also has proteolytic digestive enzymes, potent antimicrobial substances [34], prophenoloxidase cascade, and immune molecules such as agglutinins which help in expression of immune effector genes and mediating communication between the gut and the fat body contents when microbes are encountered [48]. The presence of all these immune molecules can prevent establishment and maturation of* T. congolense* and* T. brucei* as these parasites use midgut in part of their lifecycles; hence the low infections rates are detected. It is also known that* T. congolense* subtype parasites are generally low with low parasitaemia compared to those of* T. vivax* [38]. It is therefore possible that such scanty parasites are rarely picked up by the flies during blood meals which may account for the inability of the parasite to get established in the arthropod vector.

This study revealed more infection rates in males than in females. In Nigeria, higher infection rates in males than females were also reported [49]. Some studies suggest that female flies should have higher infection rates than males as they live longer than males and thus they have higher chances of getting infection [50]. However, this relationship has not been established. In contrast, other studies explain that more males may be infected than females as they are involved in sex activities and competition than females [51]. Whether more males or females are infected in field tsetse populations, this study found no statististical difference with variation in sex.

Apparent densities were strongly positively correlated with infection rates (*r* = 0.95). A similar observation was reported in Mouhoun River basin, Burkina Faso, where infection rates positively correlated with apparent densities (*r* = 0.97) [1]. This observation validates this parameter as an indicator of AAT transmission risk.

## 5. Conclusion

This study attests that tsetse plays a major role in maintaining the trypanosome parasite and concludes that sustainable reduction of tsetse from Mtito Andei Division can greatly reduce the prevalence of AAT. This study also showed that the host factors are equally important in epidemiology of AAT as higher trypanosome infection rates were recorded in* G. longipennis* than* G. pallidipes* but the sex of the vector is less important. Further, the study showed that apparent density of the vector is a good indicator of AAT transmission risk. The study therefore affirms the importance of designing control strategies targeting the biological vector (tsetse), mechanical vectors (*Tabanus* and* Stomoxys* spp.), and the trypanosome parasites.

## 6. Recommendations

1. Since both* T. congolense* and* T. vivax* are pathogenic in cattle and other ruminants, with the latter causing acute hemorrhagic syndrome [52], it would be important to conduct large scale integrated tsetse control in Mtito Andei by application of vector control strategies coupled with curative treatment of livestock as these animals could act as source of reinfection to tsetse.
2. Surveillance studies to determine trypanosome prevalence in livestock should be conducted in this area for effective trypanosomiasis control programs.
3. Entomological surveys should be conducted at different seasons to understand seasonal dynamics of the vectors and the associated trypanosomiasis risk. Data on seasonal variations of potential vectors can be integrated into epidemiological models to facilitate better understanding of the relative importance of cyclical and mechanical vectors in Mtito Andei.

## Figures and Tables

**Table 1:** Sequence of primers.

	Primer ID	Primer sequence (5′-3′)
Outer primers	ITS 1	GAT TAC GTC CCT GCC ATT TG
	ITS 2	TTG TTC GCT ATC GGT CTT CC

Inner primers	ITS 3	GGA AGC AAA AGT CGT AAC AAG G
	ITS 4	TGT TTT CTT TTC CTC CGC

**Table 2:** Documented band sizes of trypanosome species [19].

Trypanosome species	Expected band sizes in base pairs (bp)
*T. congolense *forest	1513
*T. congolense *Kilifi	1422
*T. congolense *Savannah	1413
*T. congolense *Tsavo	954
*T. brucei*	1207–1224
*T. vivax*	611

**Table 3:** Trypanosome infection rates in *Glossina *species.

*Glossina* species	Infection rates			Overall infection rates (%)	Total
	Uninfected	*T. vivax*	*T. congolense *forest		
*G. pallidipes*	49	2 (3.85%)	1 (1.92%)	3 (5.77%)	52
*G. longipennis*	20	6 (23.08%)	0 (0%)	6 (23.08%)	26
Total	69	8 (10.26%)	1 (1.28%)	9 (11.53%)	78

**Table 4:** Comparison of infection rates in male and female flies.

Sex	Infection rates			Overall infection rates (%)	Total
	Uninfected	*T. vivax*	*T. congolense *forest		
Male	37	5 (11.63%)	1 (2.33%)	6 (13.95%)	43
Female	32	3 (8.57%)	0 (0%)	3 (8.57%)	35
Total	69	8 (10.26%)	1 (1.28%)	9 (11.53%)	78
